# Interpretable Multi-Cancer Early Detection Using SHAP-Based Machine Learning on Tumor-Educated Platelet RNA

**DOI:** 10.3390/diagnostics15172216

**Published:** 2025-09-01

**Authors:** Maryam Hajjar, Ghadah Aldabbagh, Somayah Albaradei

**Affiliations:** 1 Computer Science Department, Faculty of Computing and Information Technology, King Abdulaziz University, Jeddah 23218, Saudi Arabia; galdabbagh@kau.edu.sa (G.A.); salbaradei@kau.edu.sa (S.A.); 2 Center of Research Excellence in Artificial Intelligence and Data Science, King Abdulaziz University, Jeddah 21589, Saudi Arabia

**Keywords:** MCED, TEPs, cfRNA, SHAP, XAI, biomarker discovery, interpretable machine learning

## Abstract

**Background**: Tumor-educated platelets (TEPs) represent a promising biosource for non-invasive multi-cancer early detection (MCED). While machine learning (ML) has been applied to TEP data, the integration of explainability to reveal gene-level contributions and regulatory associations remains underutilized. This study aims to develop an interpretable ML framework for cancer detection using platelet RNA-sequencing data, combining predictive performance with biological insight. **Methods**: This study analyzed 2018 TEP RNA samples from 18 tumor types using seven machine learning classifiers. SHAP (Shapley Additive Explanations) was applied for model interpretability, including global feature ranking, local explanation, and gene-level dependence patterns. A weighted SHAP consensus was built by combining model-specific contributions scaled by Area Under the Receiver Operating Characteristic Curve (AUC). Regulatory insights were supported through network analysis using GeneMANIA. **Results**: Neural models, including shallow Neural Network (NN) and Deep Neural Network (DNN) achieved the best performance (AUC ~0.93), with Extreme Gradient Boosting (XGB) and Support Vector Machine (SVM) also performing well. Early-stage cancers were predicted with high accuracy. SHAP analysis revealed consistent top features (e.g., SLC38A2, DHCR7, IFITM3), while dependence plots uncovered conditional gene interactions involving USF3 (KIAA2018), ARL2, and DSTN. Multi-hop pathway tracing identified NFYC as a shared transcriptional hub across multiple modulators. **Conclusions**: The integration of interpretable ML with platelet RNA data revealed robust biomarkers and context-dependent regulatory patterns relevant to early cancer detection. The proposed framework supports the potential of TEPs as a non-invasive, information-rich medium for early cancer screening.

## 1. Introduction

Early detection of cancer is critical for improving patient outcomes, as it enables timely intervention before the disease advances. Conventional screening methods, however, are typically limited to the detection of a single cancer type. These methods are often invasive, expensive, and not easily accessible, which reduces their feasibility for large-scale, routine application. In response to these limitations, there is increasing interest in multi-cancer early detection (MCED) strategies. MCED tests utilize non-invasive methods to detect multiple cancer types at early stages from a single biological sample, most commonly blood [1].

MCED frameworks are enabled by the increasing availability of liquid biopsy technologies, which capture tumor-related signals from circulating DNA, RNA, proteins, or other extracellular vesicles. Among these, tumor-educated platelet (TEP) RNA offers several advantages. TEPs actively incorporate tumor-derived signals through alternative splicing and RNA transfer, making them a dynamic and reflective medium of cancer presence and phenotype. Compared to cell-free DNA, TEP RNA exhibits higher transcriptomic richness, better stability in circulation, and the capacity to reveal both the tumor signal and its functional state, such as immune modulation or metabolic shifts [2].

Several efforts have investigated interpretability in omics-based cancer detection. Most studies rely on tissue-derived gene expression or methylation data from The Cancer Genome Atlas (TCGA) or Gene Expression Omnibus (GEO), which provide high-dimensional input but lack the non-invasive advantages of liquid biopsies [3,4,5,6,7,8,9,10,11]. In contrast, only a few have leveraged blood-derived data, such as serum miRNAs [12], PBMC transcriptomes [13], serum exosomes [14], or cfDNA methylation [15]. From a modeling perspective, both tree-based algorithms [3,4,6,12,13,14,15] and deep learning architectures [5,8,9,10,11] are commonly applied to capture the complexity of transcriptomic and epigenomic profiles. Among the most recent deep learning studies, the CrossNN framework [10] demonstrated robust pan-cancer classification across diverse methylome platforms with interpretable feature-level analysis, while MOGKAN [11] integrated mRNA, miRNA, and DNA methylation using a graph-based Kolmogorov–Arnold network to provide accurate and biologically meaningful predictions.

Building on these modeling frameworks, a range of interpretability techniques have been adopted to make predictions more transparent. SHAP (Shapley Additive Explanations) has become particularly prominent across studies [4,6,8,9,13,14]. Other strategies include Local Interpretable Model-Agnostic Explanations (LIME) [4,7], attention mechanisms [5,11], and network-based feature prioritization methods [12]. These explainability approaches are frequently coupled with biological validation through pathway enrichment analysis or alignment with established cancer biomarkers [5,6,7,8,9,10,11,12,13,14].

Despite the growing number of MCED studies using omics data, most focus on cfDNA methylation or protein panel-based approaches, with limited exploration of RNA. Moreover, they focus heavily on performance without adequately addressing biological interpretability. As shown in prior literature, including our own recent review [16], explainability remains a critical missing layer in MCED development.

To the best of our knowledge, this is the first study to apply explainable machine learning to TEP RNA for multi-cancer early detection. By integrating SHAP-based feature attribution, model consensus, dependence analysis, and network-level validation, this framework advances both the performance and interpretability of ML-driven cancer diagnostics using blood-derived transcriptomics.

## 2. Materials and Methods

### 2.1. Data Acquisition and Cohort Composition

This study utilized the publicly available platelet RNA-sequencing dataset published by In ’t Veld et al. [17], accessible through GEO under accession number GSE183635. The dataset comprises 2351 samples, including 1628 cancer patients spanning 18 distinct tumor types and 723 control individuals.

To maintain a clean biological contrast and improve interpretability, 333 control samples associated with chronic inflammation, systemic disease, or cancer-like conditions were excluded. These individuals were previously flagged in the original study for exhibiting confounding molecular activity that could introduce biological noise due to immune responses or prior malignancies. After exclusion, the final dataset consisted of 2018 samples, of which 1628 were cancer and 390 were asymptomatic non-cancer controls.

The data used for this analysis were extracted from the provided TEP_count_matrix and clinical_metadata files, consistent with the original publication’s preprocessing. Only protein-coding genes were retained. Samples with missing or ambiguous labels were discarded. Class balancing and model interpretation steps were performed exclusively on this cleaned dataset.

To further establish robustness, we performed external validation using an independent dataset (GSE68086, *n* = 285) from the NCBI GEO repository. To avoid data leakage, we identified 30 samples that overlapped with the primary dataset (GSE183635) and excluded them. The final external cohort thus comprised 255 unique samples. Preprocessing was harmonized by restricting analysis to the intersecting genes present in both datasets and applying the same transformations (scaling parameters) as fitted on the training cohort.

### 2.2. Preprocessing and Feature Selection

We began from the curated expression matrix (TEP_count_matrix.RData) published by In ’t Veld et al. [17], which contained normalized platelet RNA-seq data from cancer patients and non-cancer controls. This dataset had undergone library-size normalization as part of the original thromboSeq pipeline and included only protein-coding genes.

To reduce dimensionality and retain biologically relevant features, we applied a three-stage feature selection procedure:

- Statistical Filtering: One-way ANOVA was applied on 33% of the full dataset (mimicking the discovery phase of the original study), and genes with a false discovery rate (FDR) below 0.001 were retained. A maximum cap of 300 genes was imposed to limit model complexity.
- Correlation Filtering: From the ANOVA-filtered genes, we excluded features with pairwise Pearson correlation |*r*| > 0.8 to reduce multicollinearity.
- Standardization: All input features were scaled using z-score normalization within each cross-validation fold to avoid data leakage.

To address potential demographic and clinical biases, we developed a constrained optimization-based stratification method to split the dataset into 80% training and 20% testing. We used a sequential least squares programming (SLSQP) solver to minimize divergence in the distribution of four key covariates: age, sex, cancer type, and institution. This ensured demographic parity between the training and test sets while maintaining a fixed number of samples per set.

Finally, to address class imbalance during training, we applied the Synthetic Minority Oversampling Technique (SMOTE) on the training fold within each cross-validation loop, ensuring balanced representation of cancer and control samples during model learning.

### 2.3. Machine Learning Pipeline

Following feature selection, we developed a machine learning pipeline to evaluate the predictive performance of multiple classifiers in distinguishing cancer from non-cancer cases. These classifiers include Shallow Neural Network (NN), Deep Neural Network (DNN), Extreme Gradient Boosting (XGB), Support Vector Machine (SVM), Random Forest (RF), Logistic Regression (LR), and Decision Tree (DT). The final dataset was split into training (80%) and testing (20%) sets using the stratified optimization procedure described in Section 2.2. The training set was used for model development and cross-validation, while the test set was held out for final evaluation.

To address class imbalance, the Synthetic Minority Over-sampling Technique (SMOTE) was applied to the training set only, ensuring that control (non-cancer) samples were upsampled to match the number of cancer cases. Importantly, this resampling was applied within each fold of cross-validation, preventing any data leakage into the evaluation folds.

Each model pipeline also included feature standardization using a StandardScaler, which was fitted within each fold to prevent information leakage. Both SMOTE and scaling steps were encapsulated within an imbalanced-learn Pipeline object to ensure consistent and leak-proof preprocessing across all models.

We benchmarked seven machine learning classifiers grouped by model family:

- Tree-based models: DT, RF, and XGB.
- Linear and non-linear models: LR and SVM.
- Neural models: Shallow NN and DNN.

The shallow NN consisted of a single hidden layer with ReLU-activation followed by a dropout layer. DNN used the same architecture with an additional hidden layer and a second dropout layer. Both models used sigmoid activation in the output layer for binary classification and were trained using the Adam optimizer with binary cross-entropy loss. Detailed parameters for both NN and DNN are given in Table 1.

All seven models were optimized using 5-fold stratified cross-validation on the training set. Hyperparameter tuning for all models was performed using a combination of randomized search and manual refinement. The final configuration for each model was selected based on Area Under the Receiver Operating Characteristic Curve (AUC) performance across cross-validation folds, after which the model was retrained on the full training set and evaluated on the held-out test set.

Threshold calibration was performed using Youden’s J statistic, computed on the training set. This method selects the decision threshold that maximizes the difference between true positive rate and false positive rate (i.e., sensitivity + specificity − 1), ensuring a balanced trade-off between sensitivity and specificity. The resulting threshold was then fixed and applied to test set predictions to ensure unbiased evaluation.

Model performance was evaluated using standard classification metrics including AUC, accuracy, sensitivity (recall), specificity, precision, and F1-score. These metrics were calculated on the held-out test set to assess generalization performance. Confusion matrices and full classification reports were also generated to support detailed error analysis and class-level insight.

### 2.4. SHAP Explainability Framework

To interpret the contribution of individual features to model predictions, we applied SHAP, a unified framework for model-agnostic and model-specific interpretability. SHAP assigns each feature a contribution value reflecting its impact on the prediction for a given sample, grounded in cooperative game theory.

We used the appropriate SHAP explainer based on model type:

- TreeExplainer for tree-based models (DT, RF, XGB),
- LinearExplainer for LR,
- KernelExplainer for SVM,
- DeepExplainer for neural networks (NN and DNN).

SHAP values were computed for the positive class (cancer prediction) using the entire training set as background for all models except SVM. Due to the computational cost of KernelExplainer, a representative subset of 50 training samples (selected via KMeans clustering) was used as background for SVM, and only 50 test samples were evaluated.

To enhance interpretability and minimize the influence of spurious extreme SHAP values, we applied outlier filtering per gene (feature). This was done using an adaptive IQR-based filtering strategy, where SHAP values outside dynamic bounds (based on the feature’s interquartile range) were zeroed out. If no acceptable IQR multiplier produced a filtering ratio between 5% and 10%, a percentile clipping fallback (e.g., keeping only values between the 1st and 99th percentiles) was applied instead. Filtered SHAP values were used for global interpretation, while unfiltered values were retained for local explanation plots (Supplementary Figure S1 illustrates both approaches). Interpretation outputs included:

- Global feature importance: SHAP bar plots and beeswarm plots per model.
- Local explanations: Force plots highlighting gene-level impact on individual predictions.
- SHAP dependence plots: To visualize non-linear or interaction effects between gene pairs.

For each model, dependence plots were generated for the top three features ranked by their mean absolute SHAP values. Two versions were created for each: one using all test samples and another excluding samples whose SHAP values had been zeroed out through the outlier filtering procedure, to emphasize informative variation. For plots without an explicitly defined interaction feature, SHAP’s default behavior was applied, which automatically selects the gene with the strongest interaction based on internal SHAP interaction values. Additional plots were also generated using manually specified interaction features, chosen from the other top-ranked genes to allow consistent biological comparisons. All plots were saved with jitter to reduce overlap and enhance interpretability.

### 2.5. Weighted SHAP Aggregation

While individual models each highlight their own set of top-ranking genes, identifying genes that are consistently influential across models provides a more robust foundation for biological interpretation. To synthesize model outputs into a unified view, we employed a weighted SHAP aggregation strategy that integrates both the magnitude of SHAP values and the reliability of each model’s performance. This allowed us to prioritize genes not just based on isolated importance, but also in proportion to how confidently each model performs.

To produce a unified global ranking of genes across all machine learning models, we computed a weighted SHAP importance score per gene. For each model, SHAP values were first scaled (MinMax normalization), and then the average SHAP value per gene was calculated. To emphasize better-performing models, we assigned a weight to each model based on its classification AUC score on the test set. The final global importance score per gene was obtained by multiplying its scaled importance by the corresponding model weight and aggregating across all models. This approach ensures that genes ranked highly by consistently high-performing models receive greater emphasis in the overall ranking.

### 2.6. Model Agreement Analysis and Biological Relevance Evaluation

To evaluate the consistency of gene rankings between models, we computed pairwise Spearman rank correlation coefficients. For each model pair, we identified the common genes that were ranked with non-zero SHAP values by both models. We then calculated the Spearman correlation between their ranked SHAP importance values across these shared genes. The resulting pairwise correlation matrix was visualized as a heatmap, providing insight into the agreement level across models regarding gene importance. This analysis complements the consensus ranking by assessing the cross-model robustness of feature prioritization.

To evaluate biological relevance, SHAP-derived top-ranking genes were compared against known cancer-associated transcripts identified in external literature or curated cancer gene databases. This contextual validation supported interpretation of model behavior and feature importance.

## 3. Results

This study analyzed 2018 platelet RNA samples derived from the public dataset GSE183635, consisting of 1628 cancer cases and 390 asymptomatic non-cancer controls across 18 tumor types. To support model interpretability and robust evaluation, symptomatic controls were excluded to minimize biological noise and a stratified optimization strategy was employed to partition the dataset into training and testing subsets with balanced distributions of age, sex, cancer type, and data source. The overall pipeline included rigorous preprocessing, multi-model classification, and SHAP-based explainability, with a focus on identifying consistent transcript-level biomarkers for cancer detection.

The remainder of this section presents model performance and evaluation metrics (Section 3.1), stage-specific detection performance (Section 3.2), cancer-specific detection performance (Section 3.3), and SHAP-based interpretability findings (Section 3.4), including global SHAP feature importance, SHAP dependence plot analysis, local SHAP interpretation, consensus gene importance, and model agreement analysis via Spearman correlation.

### 3.1. Model Performance Evaluation

We evaluated the performance of seven machine learning classifiers on the held-out test set (*n* = 405). These included NN, DNN, XGB, SVM, RF, LR, DT. Performance was assessed using multiple metrics, including AUC, average precision (AP), sensitivity (recall), specificity, precision, accuracy, F1-score, and balanced accuracy.

**Figure 1:**
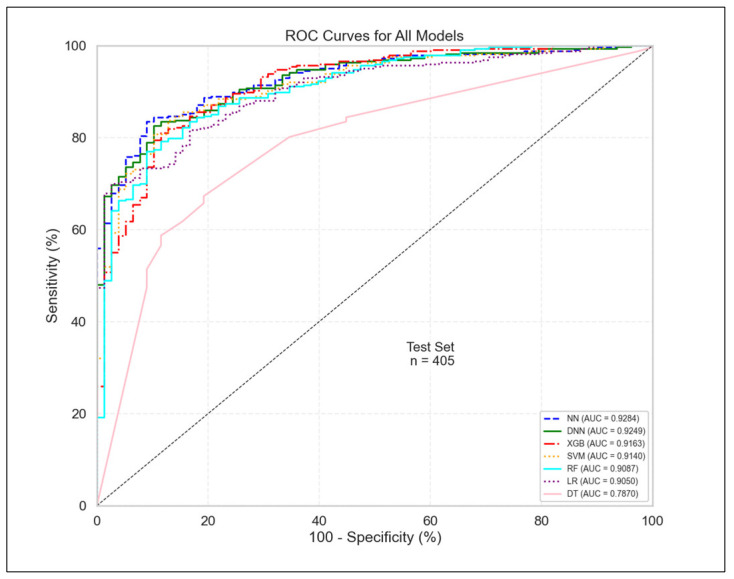
Receiver Operating Characteristic (ROC) Curves for all classifiers.

As shown in Figure 1 (ROC curves), all models except DT exhibited strong discriminative performance, with AUC values ranging from 0.905 (LR) to 0.928 (NN). The neural models (NN and DNN) achieved the highest AUCs (0.9284 and 0.9249, respectively), followed closely by XGB (0.9163) and SVM (0.9140). The DT model underperformed across all metrics, with an AUC of 0.7870 and the lowest F1-score and sensitivity, highlighting its limited generalizability in this context.

**Figure 2:**
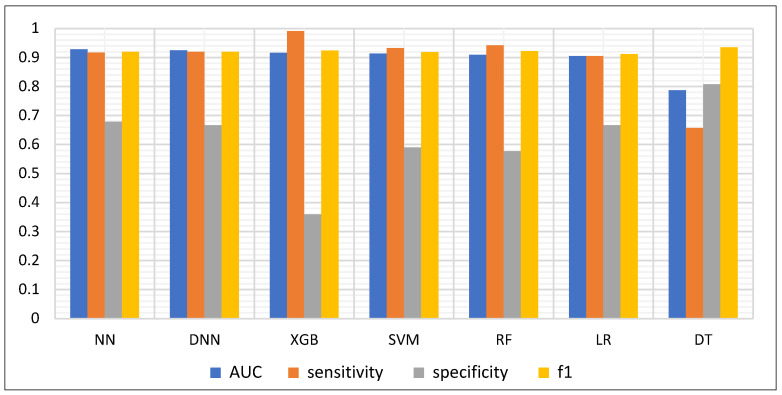
Key metrics comparison for all seven classifiers: Neural Network (NN), Deep Neural Network (DNN), Extreme Gradient Boosting (XGB), Support Vector Machine (SVM), Random Forest (RF), Logistic Regression (LR), and Decision Tree (DT). Evaluation metrics include Area Under the Receiver Operating Characteristic Curve (AUC), sensitivity, specificity, and F1-score (F1).

When comparing key metrics across all models (Figure 2), XGB achieved the highest sensitivity (0.9908) but also the lowest specificity (0.3590), suggesting a trade-off between over-detection and false positives. In contrast, DT achieved the highest specificity (0.8077) but at the cost of reduced sensitivity (0.6575). The NN and DNN models struck the most balanced trade-off; NN, for example, achieved nearly double the specificity of XGB (0.6795 vs. 0.3590) while sacrificing only 0.07 in sensitivity (0.9174 vs. 0.9908).

For the best-performing NN model, the confusion matrix revealed 300 true positives, 53 true negatives, 27 false negatives, and 25 false positives, resulting in a sensitivity of 91.74% and specificity of 67.95%. This reflects the model’s strong ability to detect cancer cases with an acceptable false-positive rate.

AP results were consistent with the AUC findings, with NN again achieving the highest score (0.9826), followed by DNN (0.9816), further supporting the reliability of the probability estimates and overall classification performance. A full breakdown of performance metrics is provided in Table 2.

### 3.2. Stage-Specific Detection Performance

**Figure 3:**
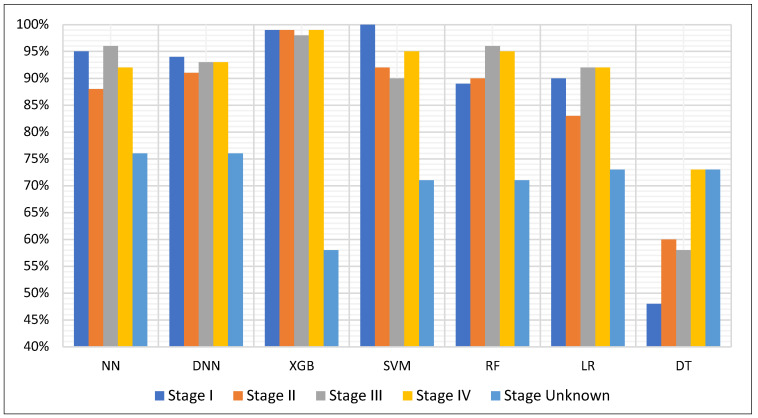
Detection accuracy per cancer stage for all seven classifiers: Neural Network (NN), Deep Neural Network (DNN), Extreme Gradient Boosting (XGB), Support Vector Machine (SVM), Random Forest (RF), Logistic Regression (LR), and Decision Tree (DT).

To further investigate the performance of each model across cancer progression, we analyzed detection accuracy per stage (I–IV and unknown), as shown in Figure 3. Overall, most models maintained high performance in detecting late-stage cancers (Stage III and IV), while varying more substantially in early-stage detection. Among all classifiers, SVM achieved the highest Stage I detection rate, reaching perfect accuracy on the limited number of early samples (*n* = 19). XGB demonstrated exceptional consistency across known stages, achieving over 98% accuracy in Stages I through IV, but suffered a substantial drop for samples with unknown stage—recording the lowest detection accuracy among all models for this subgroup, suggesting possible overfitting to clearer clinical cases.

DNN slightly outperformed NN in early-stage detection, achieving 95% and 91% accuracy for Stages I and II, respectively, versus 94% and 88% for NN. However, this 1–2% lead reversed in late-stage detection, with NN performing marginally better in Stage IV. Both neural models remained highly consistent overall, but these subtle differences may inform prioritization in early screening applications. In contrast, the DT model performed noticeably worse across all stages, particularly in early-stage detection, reinforcing its limited utility in sensitive clinical scenarios. Finally, regardless of architecture, most models exhibited a marked dip in accuracy for samples with missing or ambiguous stage labels, likely due to clinical heterogeneity or annotation uncertainty in that group.

### 3.3. Cancer-Specific Detection Performance

**Figure 4:**
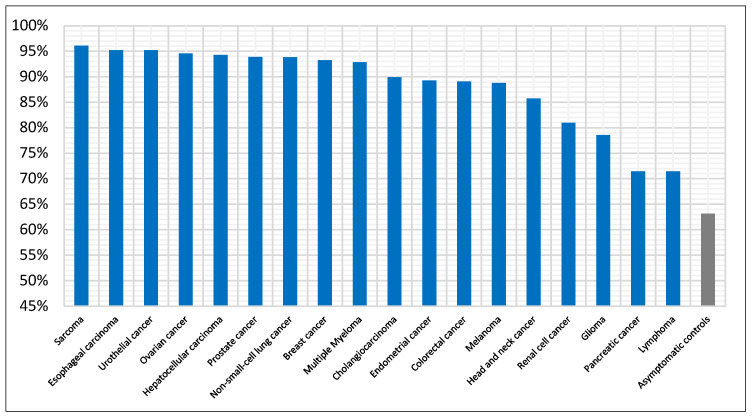
Detection accuracy per cancer type averaged across all seven classifiers. Controls are also included for reference (grey bar).

**Figure 5:**
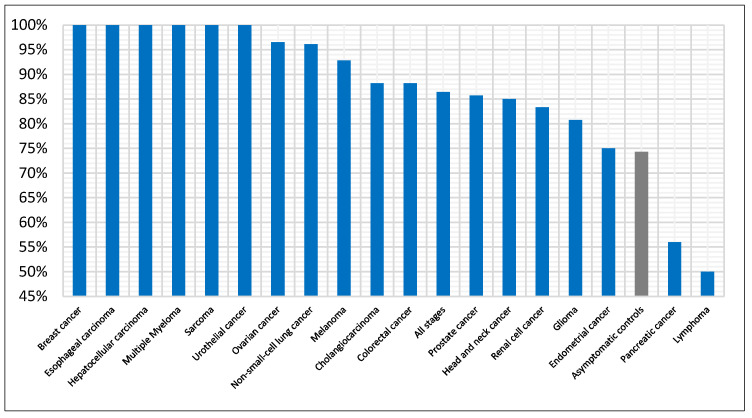
Detection accuracy per cancer type for the NN. Controls are also included for reference (grey bar).

We have also investigated the performance across cancer types. First, we evaluated the average detection accuracy per cancer type across all models and then examined the best performing model (i.e., NN) in detail. Figure 4 summarizes the mean accuracy by cancer type, averaged across all models. Thirteen cancer types are at or near 90% accuracy, and these account for 71% of all cancer test samples. Sarcoma shows the highest mean accuracy at 96%, while pancreatic cancer and lymphoma are the lowest at 71%. Control samples are also shown and have the lowest accuracy compared with cancer types at 63%. For the best performing model (Figure 5), accuracy for control samples increased to 74%. Six cancer types were detected with 100% accuracy, including sarcoma and breast cancer. Nine cancer types were in the range 81–97%, while only three were lower, between 50–75%. For performance of the other models by cancer type, see Supplementary Figures S2–S8.

### 3.4. SHAP-Based Interpretability Findings

#### 3.4.1. Global SHAP Feature Importance

**Figure 6:**
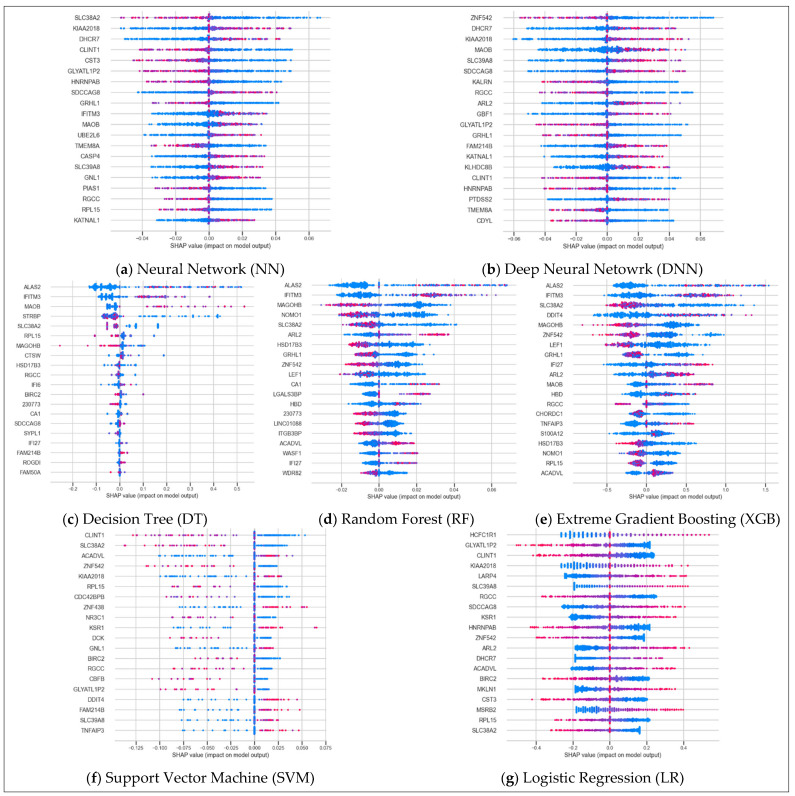
Global SHAP feature importance visualized using beeswarm plots for all seven classifiers (panels **a**–**g**). For each panel, dots represent samples, with color indicating the feature value: pink for high values and blue for low values. The horizontal position reflects the SHAP value, representing the impact of that feature on the model’s output. Note: the gene USF3 (official symbol) appears as its alias KIAA2018 in the chart.

To evaluate global explainability across models, we examined the SHAP beeswarm plots generated for each classifier (Figure 6). Our assessment focused on three interpretability criteria: (1) SHAP value dispersion, reflecting the overall spread of gene impact across samples; (2) color gradient clarity, indicating whether changes in gene expression exhibit a monotonic relationship with model output; (3) robustness, or how consistently the feature importance is distributed across samples without having gaps. These factors together provided a qualitative view of how effectively each model captured biologically meaningful patterns in the transcriptomic data.

LR exhibited the most distinct gradient, with a smooth and well-separated shift from low to high SHAP values, reflecting a clear linear relationship between feature expression and model output. While SVM also demonstrated a good gradient, its consistency and spread were weak; many samples appeared as outliers or were filtered out, limiting interpretability. Among all models, XGB had the widest spread, capturing a diverse range of SHAP values, though the gradient appeared moderate and somewhat noisy. Both RF and XGB showed moderate consistency, with distinguishable signal distributions. In contrast, the DT model showed very low consistency, with most SHAP values condensed around zero, suggesting heavy filtering or poor feature importance resolution. On the other hand, NN and DNN models revealed strong consistency with clear separation between low and high feature values. While their gradients were less linear than LR, the monotonic structure in their beeswarm plots suggests effective encoding of nonlinear relationships. Overall, NN and DNN stood out for their balance of spread, consistency, and interpretability.

#### 3.4.2. SHAP Dependence Plot Analysis: Feature Interactions

SHAP dependence plots were generated to explore feature interactions and non-linear contribution patterns across models. Among 82 plots generated, only a subset revealed biologically interpretable trends. Eight gene–gene interactions were identified across NN, RF, XGB, and LR models, highlighting both conditional activation and suppression mechanisms.

**Figure 7:**
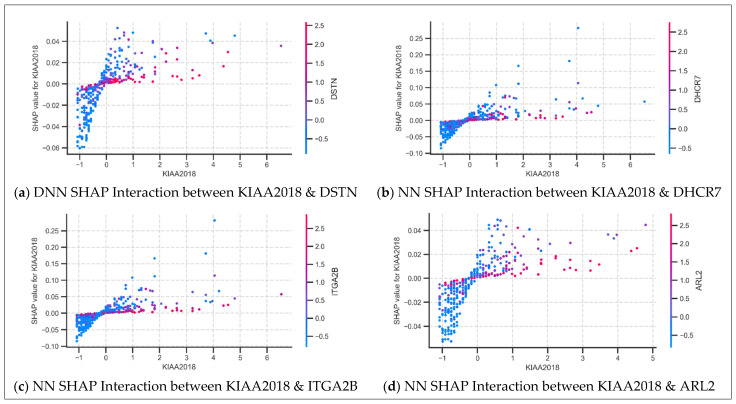
Selected SHAP dependence plots from neural models: Neural Network (NN) and Deep Neural Network (DNN), highlighting the role of context-dependent activation of KIAA2018. Each plot (panels **a**–**d**) illustrates the interaction between two genes, where each dot represents a sample and the color denotes the expression level of the second gene. The *x*-axis shows the expression level of the first gene, and the *y*-axis shows its SHAP value, indicating its contribution to the model prediction. Note: for panels (**b**,**c**), similar trends were observed in the DNN model but are omitted here for brevity. The gene USF3 (official symbol) appears as its alias KIAA2018 in the chart.

USF3 (also known as KIAA2018) consistently exhibited a pattern of context-dependent activation in the neural models, where its SHAP value increased with expression only when co-expressed genes (ITGA2B, DHCR7, ARL2, and DSTN) were expressed at low levels. This trend was observed in both NN and DNN for all interactions except ARL2 and DSTN. Specifically, the ARL2 interaction appeared only in the NN model, while the DSTN interaction was exclusive to the DNN model. In all cases, USF3′s SHAP value increases with its expression only when the interacting gene is expressed at low levels (blue). When the interacting gene is highly expressed (pink), USF3′s contribution flattens near zero, regardless of its own expression (Figure 7). This consistent conditional activation behavior suggests that USF3′s predictive influence is modulated by the expression levels of specific co-expressed genes.

**Figure 8:**
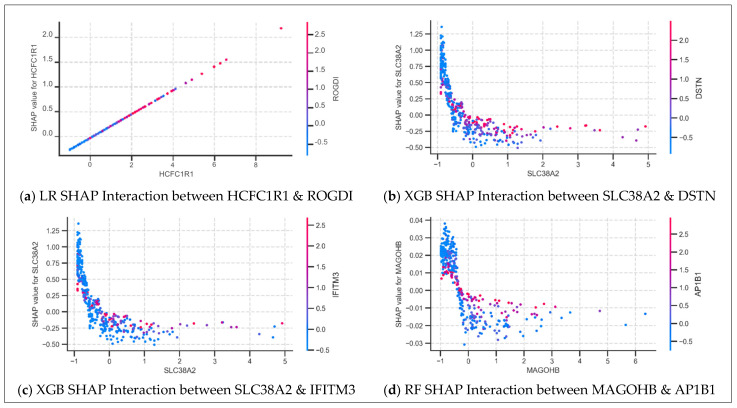
Selected SHAP dependence plots from Logistic Regression (LR), Extreme Gradient Boosting (XGB), and Random Forest (RF). Each plot illustrates the interaction between two genes, where each dot represents a sample and the color denotes the expression level of the second gene. The *x*-axis shows the expression level of the first gene, and the *y*-axis shows its SHAP value, indicating its contribution to the model prediction. The plots highlight distinct predictive patterns: (**a**) co-expression-driven behavior in HCFC1R1; and (**b**–**d**) suppression patterns combined with context-dependent activation in MAGOHB and SLC38A2.

MAGOHB and SLC38A2, derived from the RF and XGB models, respectively, both exhibit suppression patterns in which increasing gene expression leads to a sharp decline in SHAP value followed by a plateau near zero (Figure 8). However, this effect is conditional. When their interacting genes (AP1B1 for MAGOHB, and DSTN or IFITM3 for SLC38A2) are highly expressed, the SHAP values of MAGOHB and SLC38A2 remain consistently close to zero, indicating that their contributions to the model prediction are effectively suppressed. In contrast, when the interacting genes are expressed at low levels, MAGOHB and SLC38A2 regain influence, and their SHAP values reflect their own expression, often following a suppressive trend. This behavior reflects a combined mechanism of suppression and context-dependent activation. Meanwhile, HCFC1R1, based on the LR model, displays a linear positive relationship between expression and SHAP value, with a smooth gradient of ROGDI expression, indicating a co-expression-driven contribution in a linear setting. Collectively, these plots illustrate diverse gene–gene interaction mechanisms, including gating-like suppression, conditional activation, and linear co-expression. 

#### 3.4.3. Local Interpretation Comparison Across Models

**Figure 9:**
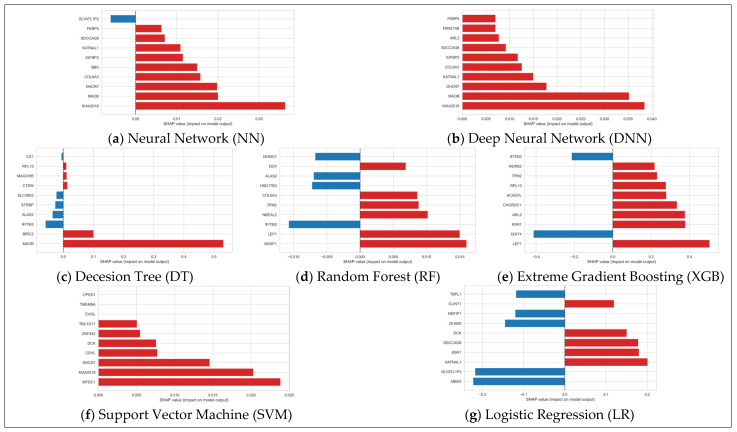
Local SHAP explanations for Sample #63 across seven models (panels **a**–**g**). Each panel displays the top ten genes contributing to the cancer prediction for this sample, selected from the top confidently predicted cancer case by both NN and DNN (prediction probability = 1.000). Red bars indicate features that increase the cancer prediction score, while blue bars indicate features that reduce it. Note: the gene USF3 (official symbol) appears as its alias KIAA2018 in the chart.

To compare gene-level contributions across models, we selected Sample #63—a confidently predicted cancer case by both NN and DNN (prediction probability = 1.000). Local SHAP explanations were generated across all seven models (NN, DNN, LR, SVM, RF, XGB, DT) to reveal how each model distributes attribution for the same prediction (Figure 9). While all models predicted cancer correctly, the set of top contributing genes varied.

Notably, USF3 (KIAA2018) and DHCR7 were highly ranked in both NN and DNN, reinforcing their shared importance in deep learning-based models. Meanwhile, COL6A3 appeared among the top contributors in NN, DNN, RF, and DT, indicating broader agreement across model families. Other genes such as GLYATL1P2 (in NN and LR), DCK (in LR, RF, SVM), and IFITM3 (in RF, XGB, DT) also showed cross-model recurrence, suggesting shared underlying patterns despite differences in model structure.

This comparison highlights the consistency of some gene-level signals across models, while also emphasizing model-specific biases in how predictions are constructed—a crucial insight for downstream biological interpretation and model trustworthiness.

#### 3.4.4. Weighted SHAP Aggregation: Consensus Gene Importance

**Figure 10:**
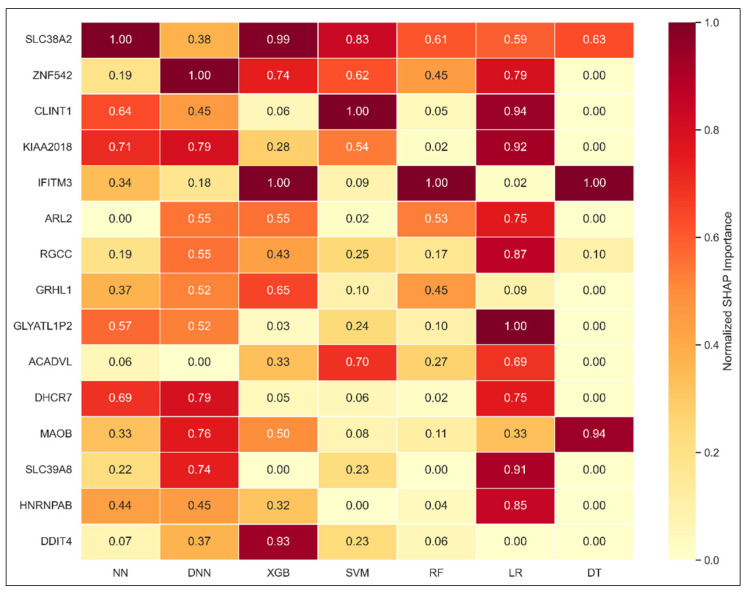
SHAP importance heatmap of top 15 consensus genes across models. Left side shows top selected genes using the weighted SHAP aggregation approach. Darker shades indicate higher relative importance within each model. DT model was excluded from the aggregation but included here for reference. Note: the gene USF3 (official symbol) appears as its alias KIAA2018 in the chart. Abbreviations: Neural Network (NN), Deep Neural Network (DNN), Extreme Gradient Boosting (XGB), Support Vector Machine (SVM), Random Forest (RF), Logistic Regression (LR), Decision Tree (DT).

To identify genes with consistently high predictive value across models, we applied a weighted SHAP aggregation strategy. SHAP values from each model were normalized individually and then weighted by the model’s classification AUC, reflecting its reliability. The top 15 genes derived from this approach were visualized in a cross-model heatmap (Figure 10), highlighting both areas of agreement and divergence.

Genes such as SLC38A2, USF3, and DHCR7 ranked among the highest across multiple models, with SLC38A2 consistently prioritized in NN, XGB, and SVM. Other genes including CLINT1, ZNF542, ARL2, and GLYATL1P2 also showed moderate-to-high importance across several models. In contrast, genes like SLC39A8, DDIT4, and GRHL1 exhibited model-specific peaks, appearing prominently in a few models while being de-emphasized in others.

Although the DT model was excluded from the aggregation due to its inconsistent SHAP behavior, it was included in the heatmap for comparison. As expected, DT displayed limited alignment with the consensus pattern, further validating its exclusion from the weighting process. Overall, the weighted SHAP approach provided a robust consensus view, emphasizing genes that were both impactful and recognized across strong-performing models.

#### 3.4.5. Model Agreement Analysis via Spearman Correlation

**Figure 11:**
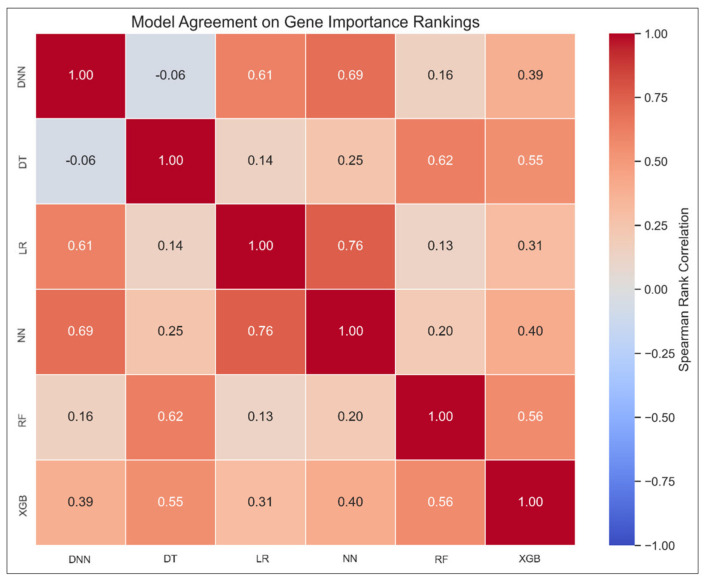
Pairwise Spearman correlation matrix of SHAP-based gene rankings across models. Each cell represents the Spearman correlation coefficient (ρ) between gene importance rankings of a model pair. Stronger correlations (dark red) indicate higher agreement in gene prioritization. DT shows limited alignment with other models, supporting its exclusion from the consensus aggregation. Abbreviations: Neural Network (NN), Deep Neural Network (DNN), Extreme Gradient Boosting (XGB), Random Forest (RF), Logistic Regression (LR), Decision Tree (DT).

To evaluate how consistently models rank gene importance, we computed pairwise Spearman correlation coefficients between the SHAP-based gene rankings of each model. The resulting correlation matrix (Figure 11) reveals several noteworthy patterns. The highest agreement was observed between the neural network models, with NN and DNN showing a strong correlation (ρ = 0.69). Interestingly, NN also aligned closely with the LR model (ρ = 0.76), suggesting convergence in feature prioritization despite architectural differences. Tree-based models such as XGB and RF showed moderate agreement (ρ = 0.56), supporting their shared role in highlighting genes like SLC38A2 and IFITM3 in the consensus analysis.

In contrast, the Decision Tree model demonstrated weak or inconsistent agreement with most other models (e.g., DT–DNN: ρ = −0.06), further validating its exclusion from the consensus ranking logic. These correlation findings underscore the robustness of the weighted consensus gene list and provide insight into which model pairs contribute overlapping versus unique biological signals.

### 3.5. Model Performance on External Validation

**Figure 12:**
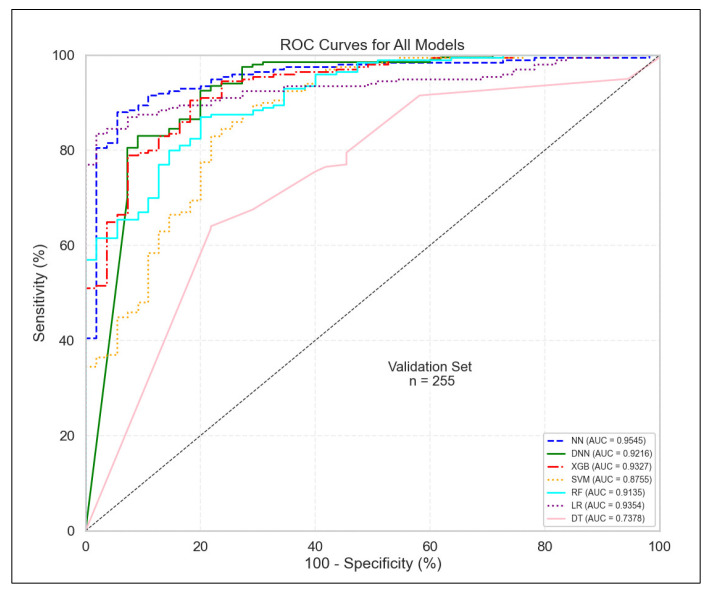
ROC curves for all classifiers in external validation cohort. Abbreviations: Receiver Operating Characteristic (ROC), Neural Network (NN), Deep Neural Network (DNN), Extreme Gradient Boosting (XGB), Support Vector Machine (SVM), Random Forest (RF), Logistic Regression (LR), Decision Tree (DT), Area Under the Curve (AUC).

Across models, performance remained robust in the external validation cohort, with AUC values comparable to those observed in the primary dataset (Figure 12). This indicates strong generalizability of the predictive models. Moreover, SHAP analysis of the external dataset revealed that 9 out of the top 15 genes overlapped with those identified in the primary dataset, namely SLC38A2, IFITM3, ZNF542, GLYATL1P2, ARL2, ACADVL, MAOB, GRHL1, and DHCR7. This reproducibility across independent cohorts supports the biological consistency of the discovered markers.

## 4. Discussion

### 4.1. Model Interpretability and Behavior

SHAP analysis revealed distinct interpretability profiles across model families. Neural networks (NN and DNN) captured rich, non-linear patterns with context-dependent feature importance, especially evident in SHAP dependence plots where gene contributions varied by the presence or expression of modulators. Tree-based models such as XGB and RF provided smoother global rankings with moderate contextual interactions, though often less sharply defined than in neural models. LR yielded interpretable, additive attributions, aligning well with global rankings but showing limited gene–gene interaction effects. SVM, using kernel SHAP, offered interpretable outputs on a smaller scale, with notable sparsity in feature attribution. DT, in contrast, demonstrated poor alignment with other models and produced limited or inconsistent SHAP signals, supporting its exclusion from consensus-based interpretation.

### 4.2. Biological Interpretation of Key Genes

#### 4.2.1. Top Predictive Genes

Among all genes analyzed, SLC38A2 emerged as the most consistently ranked and strongly cancer-associated transcript. Detected across consensus, local, and dependence SHAP interpretations, this gene encodes a glutamine transporter critical for tumor metabolism and mTOR pathway activation [18,19]. Its contribution was further reinforced by suppression interactions involving DSTN and IFITM3, highlighting its functional embedding within cancer-specific metabolic networks.

Within the top 15 consensus genes, several well-established cancer-related genes were identified. IFITM3, known for its role in immune evasion and metastasis, was detected across model types and shown to suppress the contribution of co-expressed genes [20]. ARL2, a mitochondrial GTPase, and RGCC, a cell cycle regulator, both play important roles in cellular proliferation and structural regulation [21,22]. GRHL1, a transcription factor with tumor-suppressive behavior in epithelial cancers, showed negative SHAP contributions suggestive of a protective, control-associated signature [23]. Similarly, DHCR7 (cholesterol biosynthesis) [24], DDIT4 (mTOR regulation under stress) [25], HNRNPAB (RNA splicing) [26], and SLC39A8 (zinc signaling) [27] all reflect well-documented mechanisms of tumor growth or adaptation. Additional contributors such as MAOB and ACADVL have known associations with oxidative signaling and cancer metabolism [28,29].

In contrast, among the highest-ranked genes were several with limited prior functional annotation in cancer but showing consistent model relevance, suggesting potential avenues for discovery. These include USF3 (KIAA2018), CLINT1, and ZNF542. USF3 is a putative transcription factor with low tissue specificity and expression detectable across multiple tissues, with higher levels in cerebellum and skeletal muscle [30,31]. In thyroid carcinoma, USF3 has been implicated in epithelial–mesenchymal transition (EMT) regulation, with germline compound heterozygous deletions in its polyglutamine tract proposed as risk factors for familial and sporadic thyroid cancer, particularly in Cowden syndrome-like phenotypes [32]. CLINT1, a clathrin interactor involved in vesicle trafficking and receptor endocytosis, shows broad cytoplasmic and membranous expression with low tissue specificity, including elevated RNA levels in digestive tract tissues [33,34]. It also maintains widespread expression across multiple cancers, including breast, colorectal, glioma, and pancreatic tumors, as documented in the HPA cancer atlas [34]. ZNF542, a zinc finger protein, is moderately expressed across several tissues, including brain and prostate [35,36], and has been identified as a hypermethylated locus in esophageal squamous cell carcinoma, suggesting a potential role as an epigenetic biomarker [37]. However, this role remains preliminary and hypothesis-generating, pending further validation. Taken together, these associations highlight intriguing leads that warrant future mechanistic and biomarker validation studies.

In addition to the top-ranked predictors, several medium-ranked genes (positions 16–30) demonstrated both strong biological relevance and consistent appearances in local SHAP interpretation. RPL15, a ribosomal protein implicated in chemoresistance and translation regulation, was identified across multiple models and aligns with the growing recognition of ribosomal proteins in oncogenesis [38]. KSR1, a scaffolding protein in the MAPK signaling cascade, plays a critical role in Ras-driven tumor proliferation [39]. LEF1, though slightly lower-ranked in consensus scoring, is a well-established transcription factor within the Wnt/β-catenin pathway and frequently implicated in epithelial-to-mesenchymal transition and tumor progression [40]. DCK, a deoxycytidine kinase, holds clinical importance as a biomarker of response to nucleoside-based chemotherapies such as cytarabine and gemcitabine [41].

Additionally, while lower in global rankings, several genes including COL6A3 (extracellular matrix remodeling) [42], FKBP5 (glucocorticoid signaling) [43], IGFBP2 (tumor invasion and angiogenesis) [44], and TPM2 (cytoskeletal stability) [45] appeared prominently in local SHAP plots for specific cancer samples. Their interpretability at the individual level, combined with their established roles in tumor biology, suggests they may serve as context-specific contributors or supporting biomarkers within the broader model landscape.

#### 4.2.2. Conditional Regulators and Modulators

SHAP dependence plots for USF3 (KIAA2018) revealed context-dependent behavior modulated by the expression levels of four key genes: ITGA2B, DHCR7, ARL2, and DSTN. In all cases, USF3′s predictive influence increased only when these interacting genes were expressed at low levels, suggesting conditional activation.

**Figure 13:**
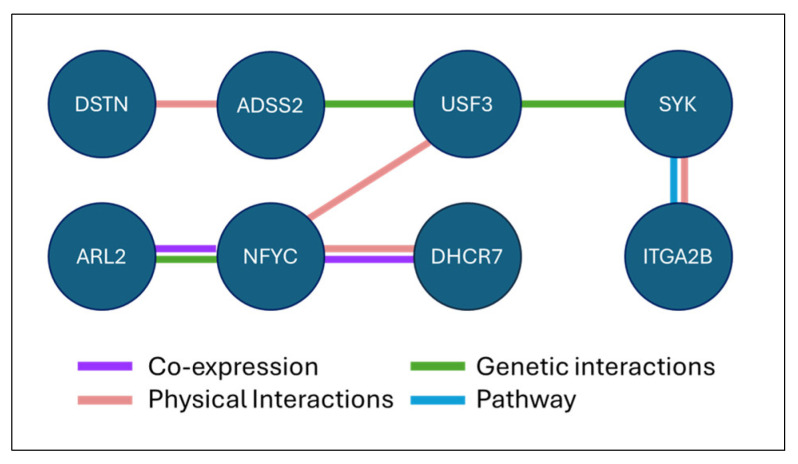
Simplified GeneMANIA network linking USF3 (KIAA2018) with SHAP-identified modulators (ITGA2B, DHCR7, ARL2, and DSTN) and intermediary nodes (NFYC, SYK, and ADSS2). Edges represent functional associations, with colors indicating the category of evidence.

To further investigate the biological plausibility of these SHAP-derived interactions, we queried GeneMANIA (version 3.6.0) [46] by pairing the USF3 gene separately with each modulator gene. Across all four runs, NFYC consistently appeared as a direct connection with USF3. Moreover, USF3 was indirectly connected to the four modulators, either through NFYC, which linked it to ARL2 and DHCR7 in a hub-like association, or through SYK and ADSS2, which linked it to ITGA2B and DSTN, respectively. These results are summarized in a simplified diagram (Figure 13), while a detailed network including all eight genes (USF3, the four modulators, and the three intermediary nodes) is provided in Supplementary Figure S9, Tables S1 and S2 for reproducibility.

NFYC is a subunit of the NF-Y transcription factor complex, which binds to CCAAT promoter motifs and regulates genes involved in processes such as cell cycle progression, lipid metabolism, and stress response [47]. In cancer, NFYC has been shown to modulate SREBF-driven pathways, linking it to the transcriptional control of metabolic genes such as DHCR7 and SLC38A2, both of which were also observed in our model [48]. Its repeated appearance as an intermediary node across multiple SHAP-modulator queries suggests that NFYC may act as a shared transcriptional regulator, potentially modulating the conditional contribution of USF3 in cancer-relevant pathways.

### 4.3. Explainability and Clinical Interpretability

ML model explainability is a critical factor for the clinical adoption of AI-driven diagnostics. In this study, SHAP provided transparent insights into the contribution of individual genes to model predictions, thereby improving confidence in outputs and highlighting biologically plausible associations. SHAP has emerged as one of the most widely adopted frameworks for interpreting complex models in biomedical research, with strengths that include model-agnostic design—applicable to tree-based algorithms, neural networks, and ensemble methods—as well as dual capacity for local interpretability at the patient level and global interpretability across datasets. Its visualization capabilities, including summary plots, dependence plots, and force plots, enhance usability for clinical audiences by making abstract statistical concepts more accessible and intuitive.

From a translational perspective, explainability is not simply a technical requirement but a prerequisite for clinical trust and adoption. SHAP contributes by clarifying decision boundaries, enabling clinicians to understand why a model predicts cancer presence or absence based on transcriptomic features. The method also supports hypothesis generation by highlighting biologically plausible gene associations that warrant experimental validation. Importantly, transparent interpretability frameworks facilitate alignment with regulatory standards, as agencies such as the FDA and EMA increasingly emphasize explainability as a criterion for approving AI-based diagnostic tools.

Explainability frameworks like SHAP also shape the trajectory of model adoption in clinical environments. By making predictions interpretable, these methods foster stronger engagement from clinicians, who are more likely to trust and integrate models that provide rationales alongside predictions. SHAP outputs strengthen interdisciplinary collaboration by bridging data scientists, molecular biologists, and healthcare professionals through a common interpretive language. Finally, interpretability enables iterative refinement of predictive systems: clinicians can flag unexpected or implausible outputs, ensuring models remain aligned with real-world clinical expectations and accelerating their pathway toward adoption in practice.

### 4.4. Methodological Contributions

This study presents a reproducible framework for interpretable cancer prediction using multi-model SHAP analysis integrated with robust machine learning evaluation. First, the dataset was carefully curated to exclude symptomatic controls, and an optimized stratification strategy was applied to ensure balanced distributions of age, sex, cancer type, and data source across training and test sets. Seven machine learning classifiers were compared across multiple evaluation metrics, revealing differences not only in predictive performance but also in interpretability profiles.

In addition to overall performance, the models were evaluated specifically on early-stage cancers, offering insight into their screening potential. SHAP values were aggregated across models using normalized absolute importance scores, weighted by AUC to emphasize high-performing classifiers. Dependence plots were employed to capture context-dependent interactions between genes, while local SHAP visualizations revealed sample-specific attribution patterns. These interactions were further validated using network tracing with tools like GeneMANIA, providing biological support for model-inferred relationships. Together, this framework offers a practical and scalable approach for feature prioritization and interpretation in omics-based cancer detection models.

Lastly, by including an independent validation cohort (GSE68086), we addressed concerns regarding overfitting and dataset-specific bias. The consistent performance across datasets and the recurrence of key genes (9 shared among the top 15) strengthens the reliability of our findings. These results highlight that the proposed models are not limited to a single dataset but exhibit potential for generalization.

### 4.5. Limitations and Future Directions

#### 4.5.1. Data Scarcity, Cohort Diversity, and Early-Stage Representation

This study benefits from the inclusion of an external validation cohort comprising 255 samples across seven cancer types. However, modest sample size, uneven cancer-type representation, and limited early-stage cases constrain the generalizability and clinical relevance of findings. Future work should increase the number of asymptomatic control samples to better delineate non-cancer baseline signatures. In addition, expanding cohort diversity with a sufficient and balanced number of samples across cancer types and stages, with particular emphasis on early-stage representation, will be critical to improving diagnostic sensitivity. Relevant trends point to multi-institutional data sharing and federated learning frameworks, which are increasingly adopted to overcome data fragmentation while preserving privacy [49].

#### 4.5.2. Domain Adaptability and Generalization

Platelet transcriptomes are influenced by environmental and technical factors that were not fully controlled in this study, which may limit model robustness across cohorts. To improve adaptability, future work should integrate complementary omics layers such as proteomics and methylomics and validate predictive markers across independent cohorts. Recent advances highlight domain adaptation approaches such as DAUD, an autoencoder-based framework validated on skin cancer histopathology across two hospitals and an external dataset, demonstrate strategies to handle institution-specific variability [50]. In addition, improving cross-population generalization requires the integration of diverse ancestry datasets. The MAGE consortium, for instance, generated RNA-seq profiles from 731 individuals across 26 global populations and showed that most gene expression and splicing variance occurs within rather than between populations, with eQTL effects largely consistent across ancestries. Such inclusive genomic resources are essential for strengthening the robustness and clinical applicability of predictive models in heterogeneous cohorts [51].

#### 4.5.3. Model Explainability and Stratified Insights

While SHAP improves interpretability, it assumes conditional feature independence, which may oversimplify relationships in biologically correlated datasets. Future work should include stratified SHAP analyses by cancer type and stage to uncover subtype- and progression-specific patterns, and extend beyond binary classification to multi-class prediction in order to distinguish shared versus unique biomarkers. Relevant trends point to the development of context-aware interpretability methods, such as Integrated Gradients and DeepSHAP, which are being applied in omics studies to better capture feature interactions.

#### 4.5.4. Functional Validation and Network Reliability

The identification of USF3 (KIAA2018), CLINT1, and ZNF542 as candidate genes in this study is based on computational predictions. While these findings highlight their potential role in cancer biology, they remain hypothesis-generating and require subsequent functional verification through dedicated in vitro and in vivo experiments. Moreover, the network-derived links (e.g., USF3–NFYC–DHCR7) should be regarded as hypothesis-generating and warrant further laboratory validation since GeneMANIA provides indirect functional associations without experimental verification,

## 5. Conclusions

This study demonstrates the potential of tumor-educated platelets (TEPs) as a powerful biosource for interpretable multi-cancer early detection using RNA-sequencing data. By integrating SHAP-based machine learning with a multi-model framework, we achieved robust classification performance across 18 cancer types while providing biologically grounded explanations at both the gene and pathway levels. Our findings uncover not only predictive biomarkers such as SLC38A2, IFITM3, and DHCR7, but also context-specific modulators and transcriptional interactions involving genes like USF3 and NFYC. The combined use of local and global SHAP interpretations, cross-model consensus, and network-level validation offers a transparent and scalable framework for biomarker discovery in liquid biopsy settings. These insights contribute to advancing both the diagnostic utility and the biological interpretability of ML-driven cancer detection and support the clinical potential of TEPs as a non-invasive, information-rich medium for early cancer screening.

## Figures and Tables

**Table 1:** Summary of Neural Network (NN) and Deep Neural Network (DNN) hyperparameters and training configurations.

Parameter	NN/DNN Configuration
Random seed	42
Input layer size	One node per feature in the dataset
No. of hidden layers	NN:1; DNN:2
Activation function	Hidden: ReLU; Output: sigmoid
Optimizer	Adam (Defaults: β1 = 0.9, β2 = 0.999, ε = 1 × 10^−7^)
Loss	Binary cross entropy
Metric	Area under the ROC curve
Early stopping—monitor	validation loss
Early stopping—patience	5
Early stopping—restore best weights	Yes
Learning Rate	Tuned in {0.001, 0.0005}.
Epochs	Tuned in {20, 30}
Batch size	Tuned in {32, 64}
Dropout rate	Tuned in {0.1, 0.3}
Hidden units	Tuned in {64, 128}
Search method	RandomizedSearchCV
No. of iterations	NN: 8; DNN: 5
Cross validation	StratifiedKFold
No. of folds	5
Shuffle	Yes
Weight initialization (Keras Default)	Kernels: Glorot uniform; Biases: zeros
Resampling method	SMOTE
Feature scaling	StandardScaler (zero mean, unit variance)
Classifier	KerasClassifier (SciKeras wrapper for Keras/TensorFlow models)
Pipeline order	(1) SMOTE; (2) StandardScaler; (3) KerasClassifier
Software	SciKeras, scikit learn, imbalanced learn

**Table 2:** Full breakdown of performance metrics for all seven classifiers: Neural Network (NN), Deep Neural Network (DNN), Extreme Gradient Boosting (XGB), Support Vector Machine (SVM), Random Forest (RF), Logistic Regression (LR), and Decision Tree (DT). Evaluation metrics: Area Under the Curve (AUC), F1-score (F1), average precision (AP), sensitivity (recall), specificity, precision, accuracy, and balanced accuracy.

Model	AUC	F1	AP	Sensitivity	Specificity	Precision	Accuracy	Balanced Accuracy
NN	0.928	0.920	0.983	0.917	0.679	0.923	0.872	0.798
DNN	0.925	0.920	0.982	0.920	0.667	0.920	0.872	0.794
XGB	0.916	0.924	0.977	0.991	0.359	0.866	0.869	0.675
SVM	0.914	0.919	0.978	0.933	0.590	0.905	0.867	0.761
RF	0.909	0.922	0.975	0.942	0.577	0.903	0.872	0.759
LR	0.905	0.912	0.977	0.905	0.667	0.919	0.859	0.786
DT	0.787	0.772	0.923	0.657	0.808	0.935	0.686	0.733

## Data Availability

The original data presented in the study are openly available in Gene Expression Omnibus (GEO) at https://www.ncbi.nlm.nih.gov/geo/query/acc.cgi?acc=GSE183635 (accessed on 10 May 2025). No new data were generated in this study.

## Supplementary Materials

The following supporting information can be downloaded at: https://www.mdpi.com/article/10.3390/diagnostics15172216/s1, Figure S1: Examples for SHAP Filtered vs Raw SHAP Distributions; Figure S2: Detection accuracy per cancer type for NN; Figure S3: Detection accuracy per cancer type for LR; Figure S4: Detection accuracy per cancer type for SVM; Figure S5: Detection accuracy per cancer type for RF; Figure S6: Detection accuracy per cancer type for DT; Figure S7: Detection accuracy per cancer type for XGB; Figure S8: Detection accuracy per cancer type for DNN; Figure S9: GeneMANIA Network Depicting Functional Associations following a query with USF3, NFYC, SYK, ITGA2B, ADSS2, DSTN, DHCR7, and ARL2; Table S1: GeneMANIA Output—Interactions; Table S2: GeneMANIA Output—Networks.

## Abbreviations

The following abbreviations are used in this manuscript:

Anova	Analysis of Variance
AP	Average precision
AUC	Area Under the Receiver Operating Characteristic Curve
CCAAT	Cytosine–Cytosine–Adenosine–Adenosine–Thymidine
cfDNA	Cell-Free Deoxyribonucleic Acid
cfRNA	Cell-Free Ribonucleic Acid
DNA	Deoxyribonucleic Acid
DNN	Deep Neural Network
DT	Decision Tree
EMT	Epithelial–Mesenchymal Transition
eQTL	Expression Quantitative Trait Locus
GEO	Gene Expression Omnibus
HPA	The Human Protein Atlas
IQR	Interquartile Range
LIME	Local Interpretable Model-Agnostic Explanations
LR	Logistic Regression
MCED	Multi-Cancer Early Detection
ML	Machine Learning
NN	Neural Network
PBMC	Peripheral Blood Mononuclear Cell
ReLU	Rectified Linear Unit
RF	Random Forest
RNA	Ribonucleic Acid
ROC	Receiver Operating Characteristic
SHAP	SHapley Additive exPlanations
SLSQP	Sequential Least Squares Quadratic Programming
SMOTE	Synthetic Minority Over-sampling Technique
SVM	Support Vector Machine
TCGA	The Cancer Genome Atlas
TEP	Tumor-Educated Platelets
XAI	Explainable Artificial Intelligence
XGB	Extreme Gradient Boosting
