# Supplementary material for: Interpretable Multi-Cancer Early Detection Using SHAP-Based Machine Learning on Tumor-Educated Platelet RNA
Source: Diagnostics (Basel). 2025 Sep 1;15(17):2216. doi: 10.3390/diagnostics15172216 (PMC12427754; doi:10.3390/diagnostics15172216)
Supplement: Supplementary file 1 [file diagnostics-15-02216-s001.zip › diagnostics-3815520-supplementary.pdf]

Supplementary Material for the Article

Interpretable Multi-Cancer Early Detection Using SHAP-Based Machine Learning on Tumor-Educated Platelet RNA

Maryam Hajjar [1,*],

Ghadah Aldabbagh [1]

and Somayah Albaradei [1,2]

[1] Computer Science Department, Faculty of Computing and Information Technology, King Abdulaziz University, Jeddah 23218, Saudi Arabia; galdabbagh@kau.edu.sa (G.A.); salbaradei@kau.edu.sa (S.A.)

[2] Center of Research Excellence in Artificial Intelligence and Data Science, King Abdulaziz University, Jeddah 21589, Saudi Arabia

* Correspondence: mhajjar0004@stu.kau.edu.sa

1. *Filtered vs Raw SHAP Distributions*

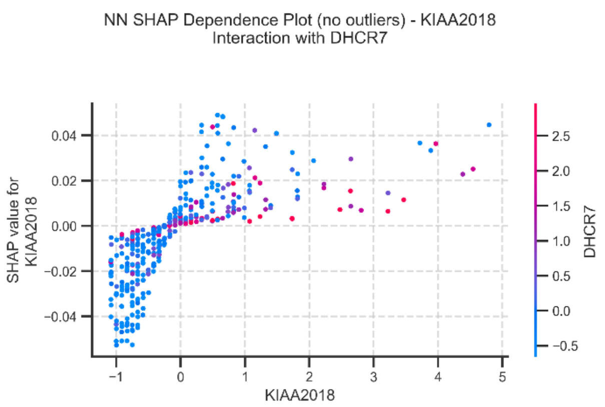

(a) Filtered dependence plot

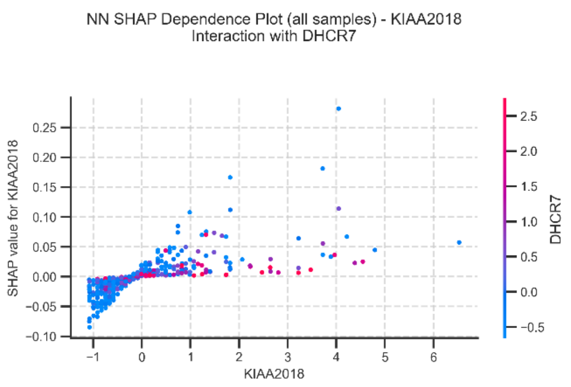

(b) Unfiltered dependence plot

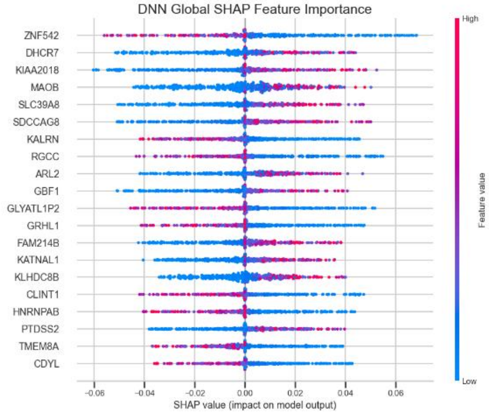

(c) Filtered Global SHAP (beeswarm)

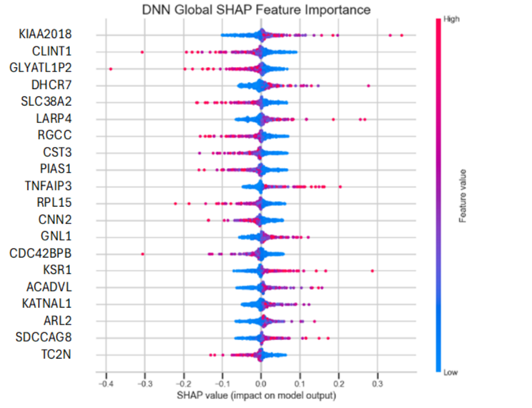

(d) Unfiltered Global SHAP (beeswarm)

Figure S1. Examples for SHAP Filtered vs Raw SHAP Distributions

This figure illustrates the effect of applying an outlier filtering strategy on SHAP values using both dependence plots (top row) and global feature importance rankings (bottom row). In the dependence plots, both the raw (b) and filtered (a) versions are presented. For interpretability, we retain the raw view in the main analyses, as it provides visibility into special cases and extreme interactions that may be biologically relevant.

For global SHAP importance, however, we report the filtered results (c) since the removal of outliers yields a more focused and stable representation of feature contributions. Outliers in the raw distribution (d) can inflate the apparent importance of certain features. For example, GLYATL1P2 appears disproportionately influential in the unfiltered plot due to extreme SHAP values, but its ranking is reduced in the filtered view, reflecting a more condensed and representative distribution of contributions. By relying on the filtered distributions for consensus explainability while still inspecting raw distributions for special scenarios, this strategy balances robustness with interpretive depth.

2. *Cancer-Specific Detection Performance*

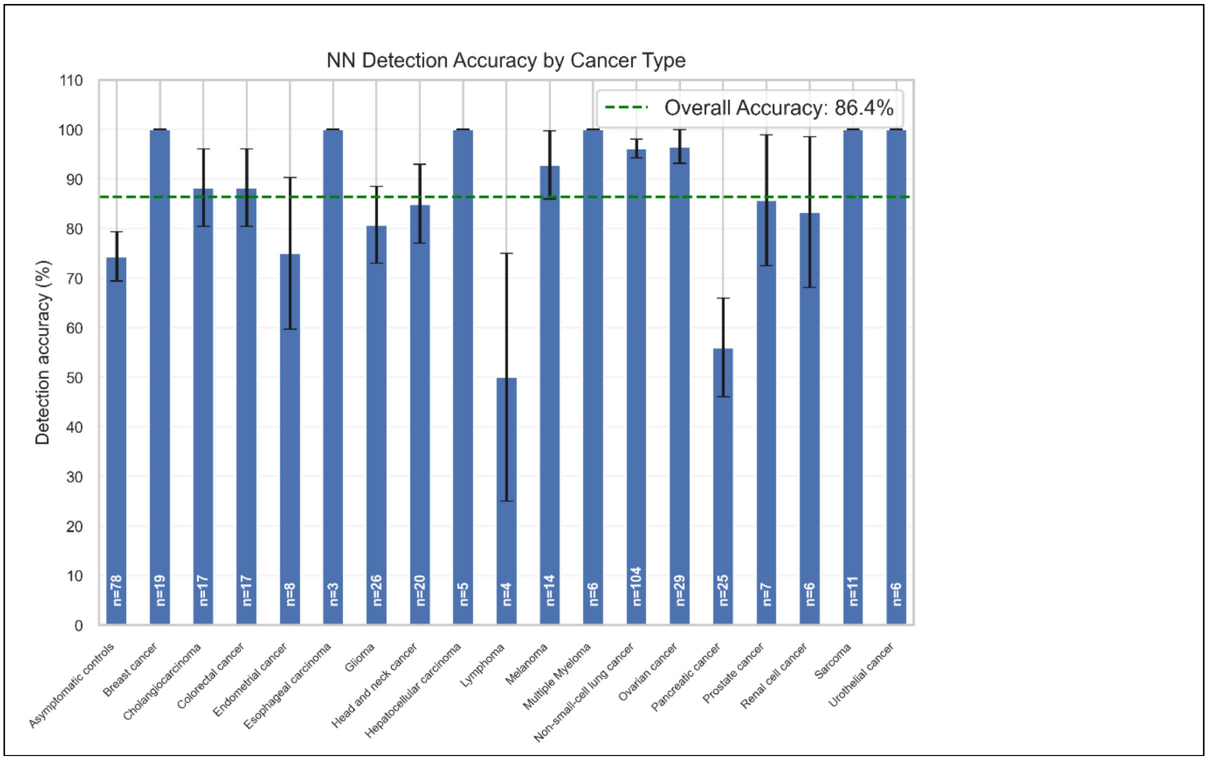

Figure S2. *Detection accuracy per cancer type for NN. Controls are also included for reference*

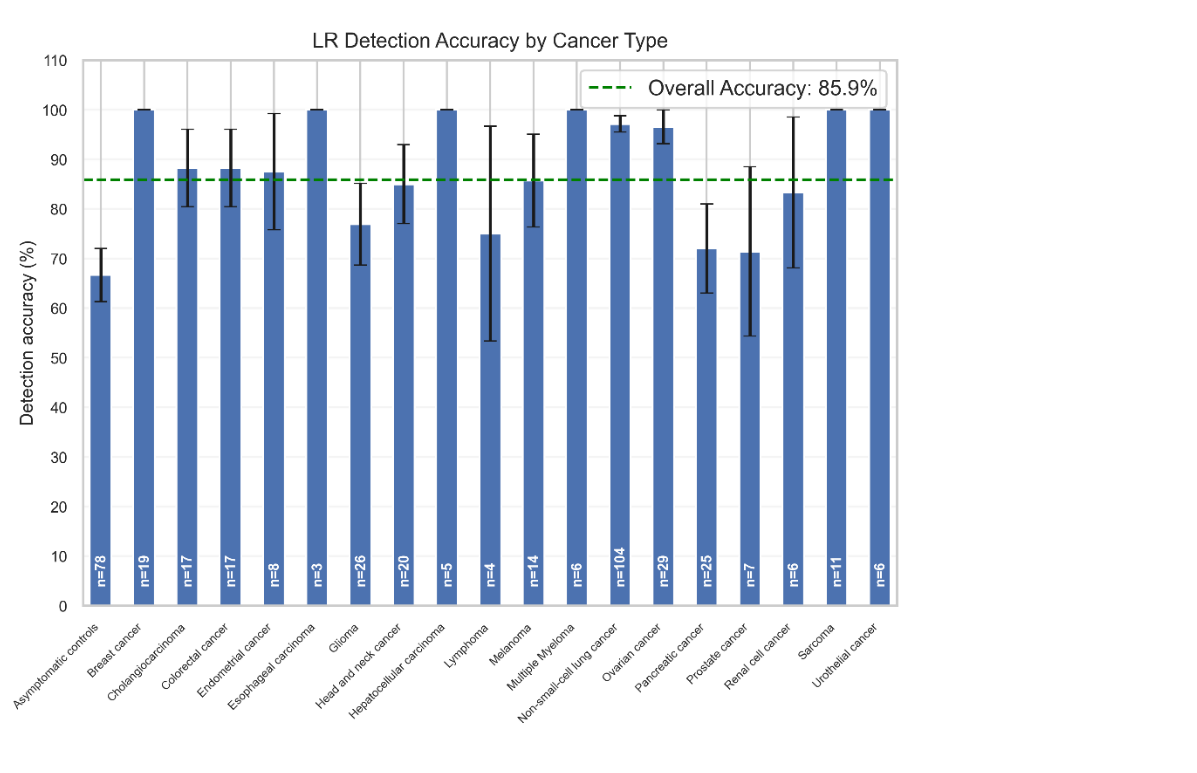

Figure S3. *Detection accuracy per cancer type for LR. Controls are also included for reference*

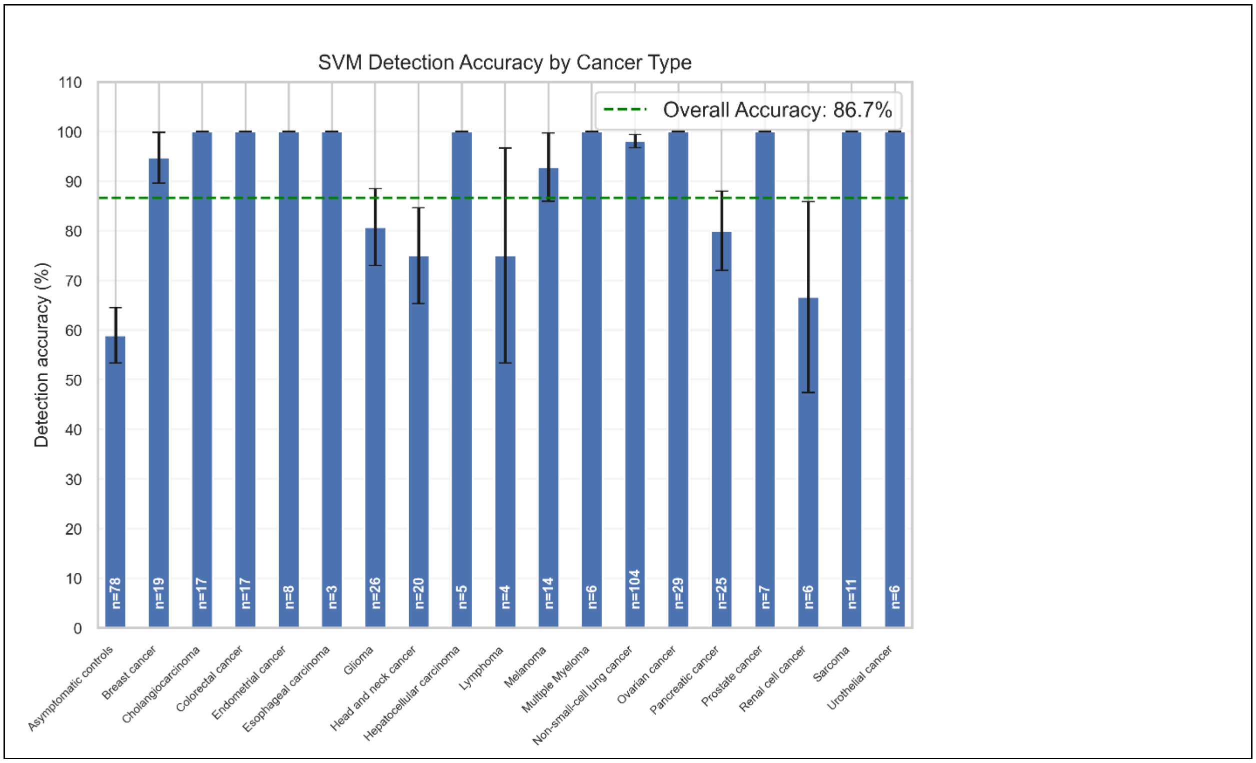

Figure S4. *Detection accuracy per cancer type for SVM. Controls are also included for reference*

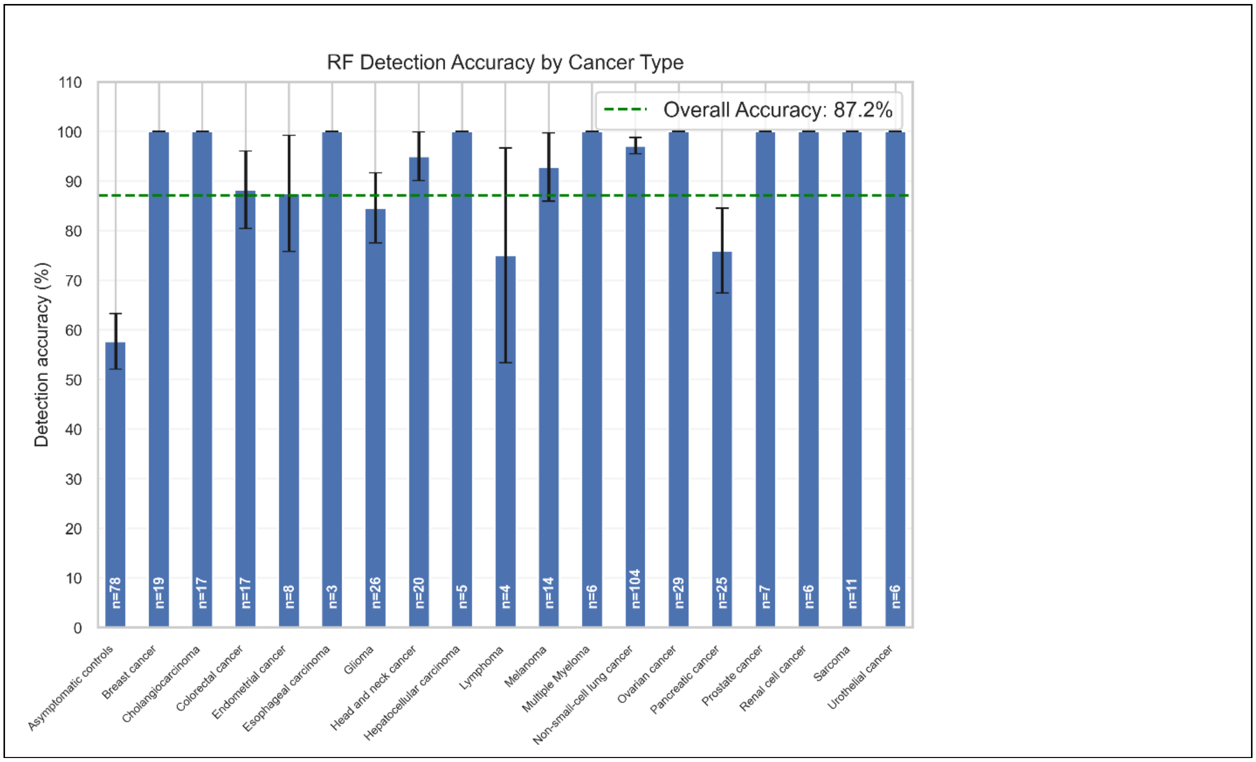

Figure S5. *Detection accuracy per cancer type for RF. Controls are also included for reference*

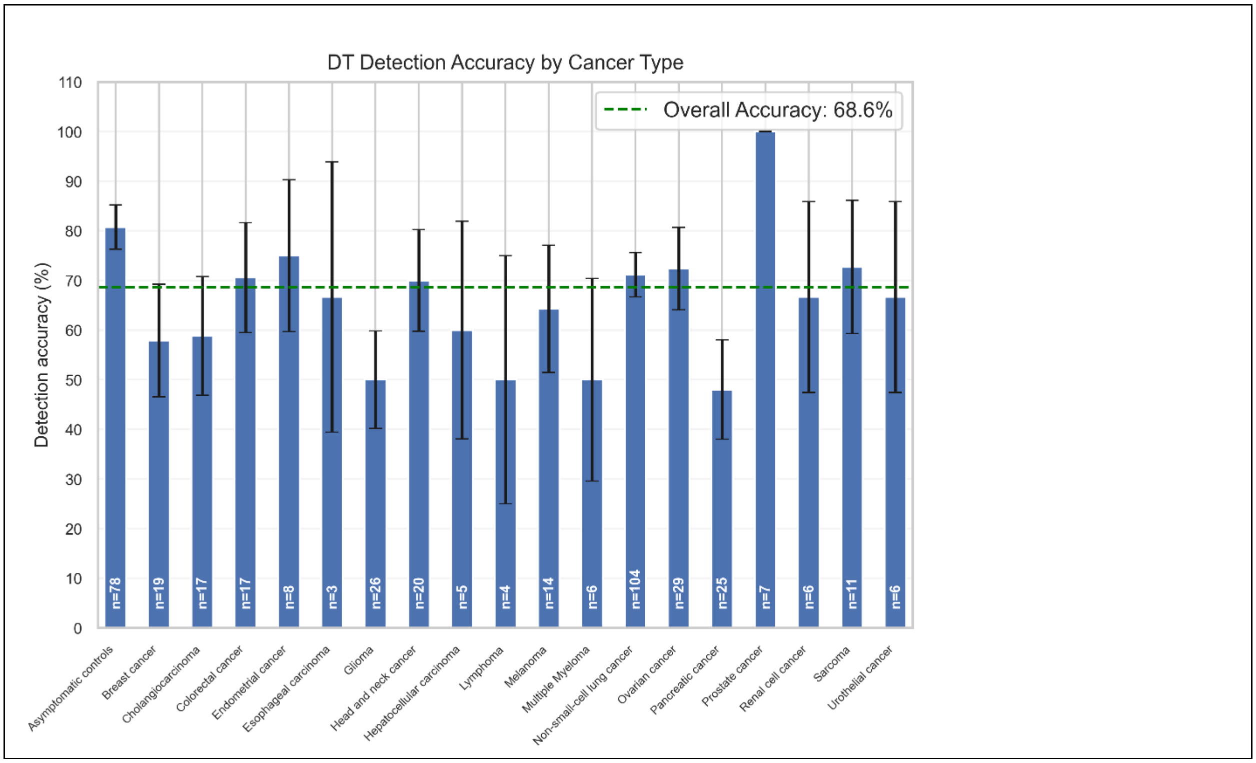

Figure S6. *Detection accuracy per cancer type for DT. Controls are also included for reference*

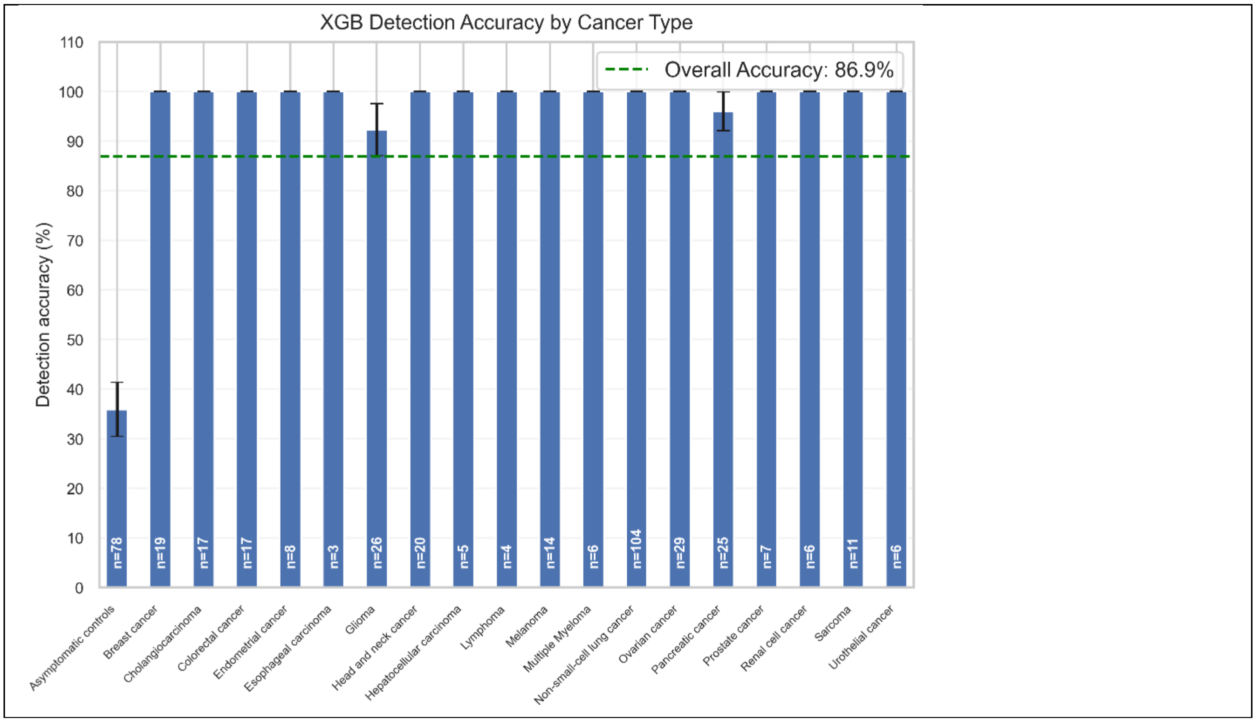

Figure S7. *Detection accuracy per cancer type for XGB. Controls are also included for reference*

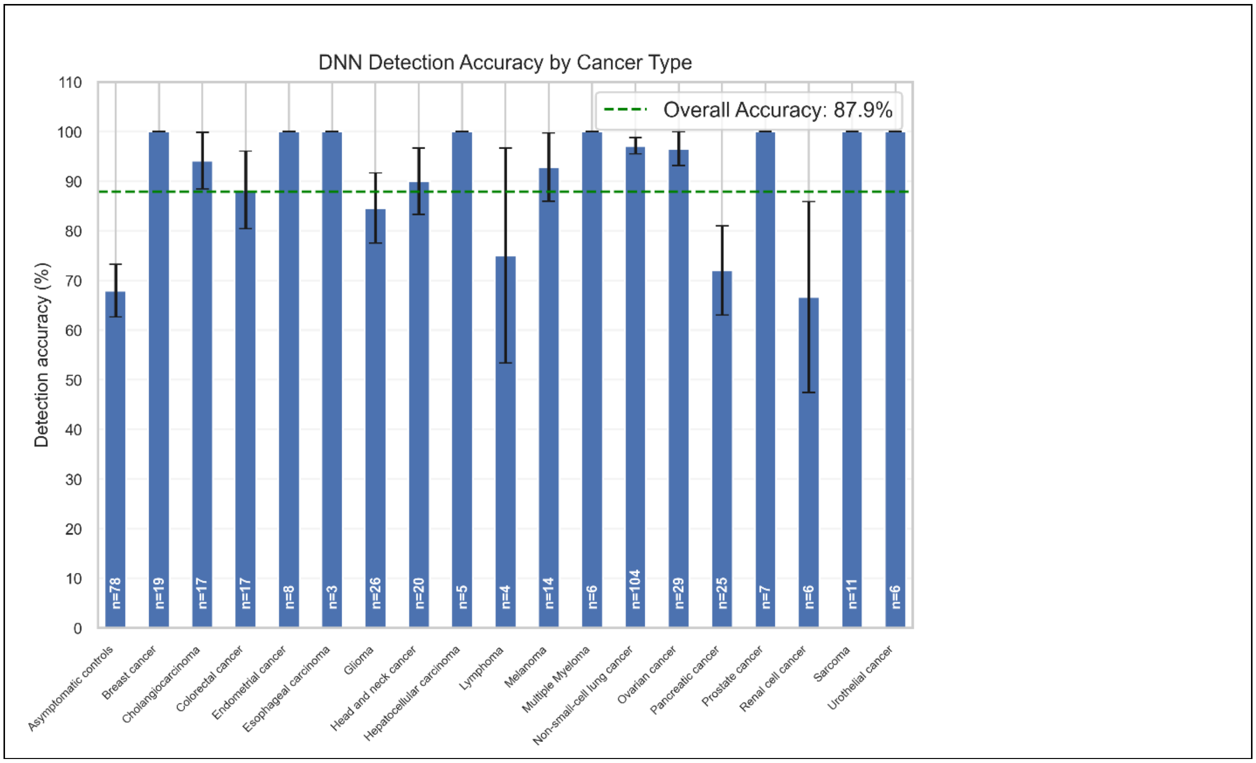

Figure S8. *Detection accuracy per cancer type for DNN. Controls are also included for reference*

3. *GeneMANIA Results*

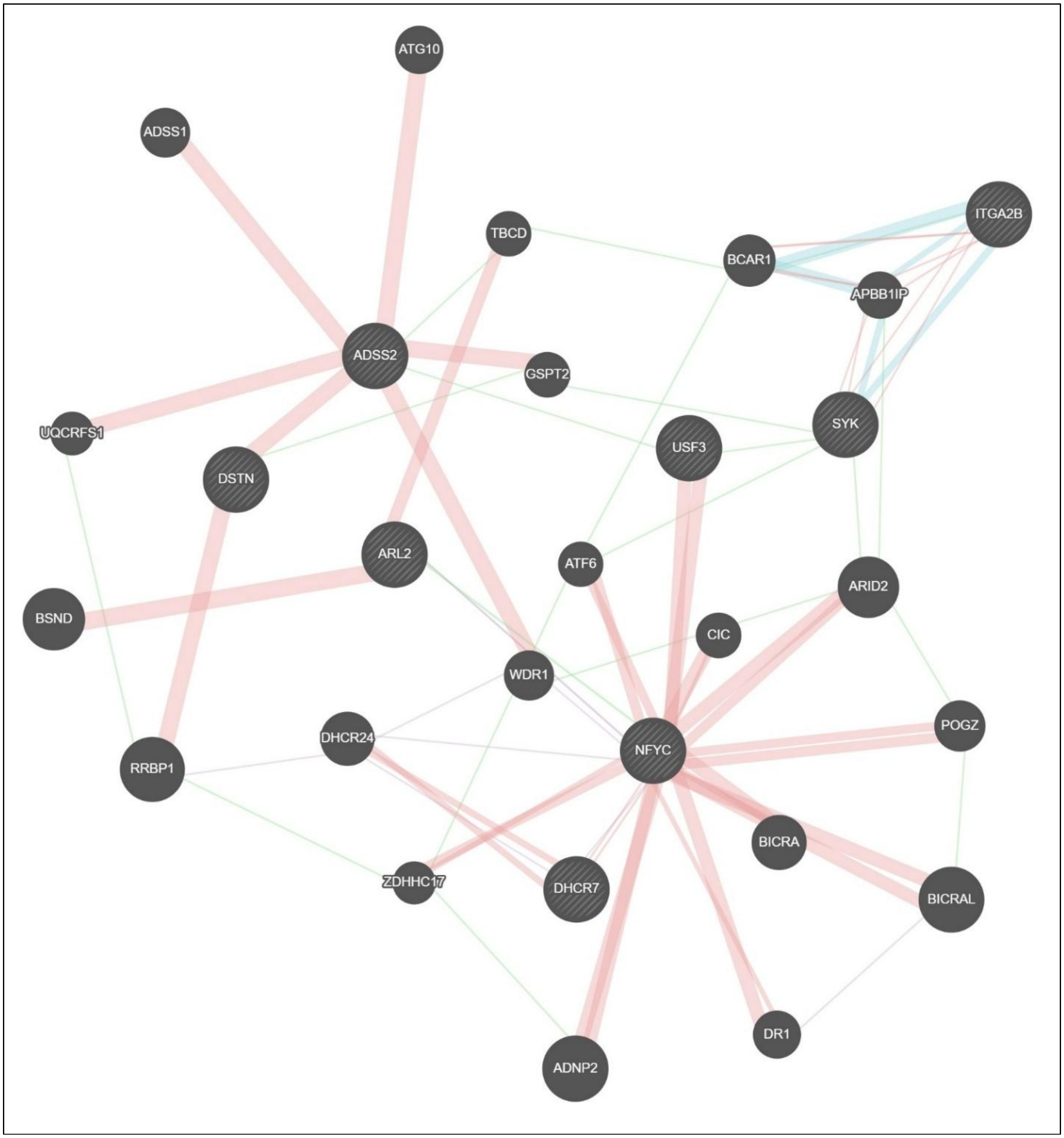

Figure S9. *GeneMANIA Network Depicting Functional Associations following a query with USF3, NFYC, SYK, ITGA2B, ADSS2, DSTN, DHCR7, and ARL2. These include the 4 genes which show dependence with USF3, USF3 it self, and 3 other genes that were determined following pair-wise GeneMania queries between USF3 and each of the 4 dependent genes.*

Table S1. *GeneMANIA Output – Interactions*

Gene 1	Gene 2	Weight	Network group	Network
**NFYC**	DHCR7	0.009356	Co-expression	Wu-Garvey-2007
**NFYC**	ARL2	0.005649	Co-expression	Wu-Garvey-2007
**DHCR24**	NFYC	0.012407	Co-expression	Wu-Garvey-2007
**WDR1**	NFYC	0.009882	Co-expression	Wu-Garvey-2007
**DR1**	BICRAL	0.015719	Co-expression	Wu-Garvey-2007
**NFYC**	ARL2	0.008354	Co-expression	Arijs-Rutgeerts-2009
**DHCR24**	DHCR7	0.012791	Co-expression	Arijs-Rutgeerts-2009
**DHCR24**	RRBP1	0.010982	Co-expression	Arijs-Rutgeerts-2009
**WDR1**	DHCR24	0.011709	Co-expression	Arijs-Rutgeerts-2009
**NFYC**	ARL2	0.035332	Genetic Interactions	Horlbeck-Gilbert-2018 A
**SYK**	USF3	0.001134	Genetic Interactions	Lin-Smith-2010
**ADSS2**	USF3	0.00241	Genetic Interactions	Lin-Smith-2010
**ARID2**	SYK	0.00036	Genetic Interactions	Lin-Smith-2010
**BCAR1**	ITGA2B	0.001545	Genetic Interactions	Lin-Smith-2010
**POGZ**	BICRAL	0.001011	Genetic Interactions	Lin-Smith-2010
**POGZ**	ARID2	0.000484	Genetic Interactions	Lin-Smith-2010
**WDR1**	ARID2	0.000359	Genetic Interactions	Lin-Smith-2010
**APBB1IP**	ARID2	0.000864	Genetic Interactions	Lin-Smith-2010
**GSPT2**	DSTN	0.005982	Genetic Interactions	Lin-Smith-2010
**GSPT2**	SYK	0.000655	Genetic Interactions	Lin-Smith-2010
**TBCD**	ADSS2	0.001421	Genetic Interactions	Lin-Smith-2010
**TBCD**	BCAR1	0.001134	Genetic Interactions	Lin-Smith-2010
**ATF6**	SYK	0.000709	Genetic Interactions	Lin-Smith-2010
**ATF6**	BCAR1	0.001202	Genetic Interactions	Lin-Smith-2010
**UQCRFS1**	RRBP1	0.00044	Genetic Interactions	Lin-Smith-2010
**ZDHHC17**	ADNP2	0.001175	Genetic Interactions	Lin-Smith-2010
**ZDHHC17**	RRBP1	0.000914	Genetic Interactions	Lin-Smith-2010
**ZDHHC17**	ATF6	0.001213	Genetic Interactions	Lin-Smith-2010
**SYK**	ITGA2B	0.128466	Pathway	REACTOME
**BCAR1**	ITGA2B	0.25993	Pathway	REACTOME
**APBB1IP**	ITGA2B	0.09483	Pathway	REACTOME
**APBB1IP**	SYK	0.117595	Pathway	REACTOME
**APBB1IP**	BCAR1	0.237934	Pathway	REACTOME
**ADSS2**	DSTN	0.236275	Physical Interactions	Rosenbluh-Hahn-2016
**WDR1**	ADSS2	0.236275	Physical Interactions	Rosenbluh-Hahn-2016
**ADSS1**	ADSS2	0.236275	Physical Interactions	Rosenbluh-Hahn-2016
**GSPT2**	ADSS2	0.236275	Physical Interactions	Rosenbluh-Hahn-2016
**UQCRFS1**	ADSS2	0.236275	Physical Interactions	Rosenbluh-Hahn-2016
**NFYC**	USF3	0.280956	Physical Interactions	Bandyopadhyay-Ideker-2010
**ADNP2**	NFYC	0.280956	Physical Interactions	Bandyopadhyay-Ideker-2010

**BICRAL**	NFYC	0.280956	Physical Interactions	Bandyopadhyay-Ideker-2010
**ARID2**	NFYC	0.280956	Physical Interactions	Bandyopadhyay-Ideker-2010
**BICRA**	NFYC	0.280956	Physical Interactions	Bandyopadhyay-Ideker-2010
**POGZ**	NFYC	0.167057	Physical Interactions	Bandyopadhyay-Ideker-2010
**DR1**	NFYC	0.280956	Physical Interactions	Bandyopadhyay-Ideker-2010
**CIC**	NFYC	0.280956	Physical Interactions	Bandyopadhyay-Ideker-2010
**ATF6**	NFYC	0.167057	Physical Interactions	Bandyopadhyay-Ideker-2010
**ZDHHC17**	NFYC	0.178961	Physical Interactions	Bandyopadhyay-Ideker-2010
**NFYC**	USF3	0.351662	Physical Interactions	IREF-spike
**ADNP2**	NFYC	0.351662	Physical Interactions	IREF-spike
**BICRAL**	NFYC	0.351662	Physical Interactions	IREF-spike
**RRBP1**	DSTN	0.457444	Physical Interactions	IREF-spike
**BSND**	ARL2	0.477157	Physical Interactions	IREF-spike
**ARID2**	NFYC	0.257332	Physical Interactions	IREF-spike
**BICRA**	NFYC	0.152304	Physical Interactions	IREF-spike
**POGZ**	NFYC	0.260247	Physical Interactions	IREF-spike
**ATG10**	ADSS2	0.482227	Physical Interactions	IREF-spike
**DR1**	NFYC	0.096526	Physical Interactions	IREF-spike
**CIC**	NFYC	0.055986	Physical Interactions	IREF-spike
**TBCD**	ARL2	0.327016	Physical Interactions	IREF-spike
**ATF6**	NFYC	0.260247	Physical Interactions	IREF-spike
**ZDHHC17**	NFYC	0.115476	Physical Interactions	IREF-spike
**SYK**	ITGA2B	0.016105	Physical Interactions	Vastrik-Stein-2007
**NFYC**	DHCR7	0.051571	Physical Interactions	Vastrik-Stein-2007
**DHCR24**	DHCR7	0.668485	Physical Interactions	Vastrik-Stein-2007
**BCAR1**	ITGA2B	0.05653	Physical Interactions	Vastrik-Stein-2007
**APBB1IP**	ITGA2B	0.046759	Physical Interactions	Vastrik-Stein-2007
**APBB1IP**	SYK	0.015623	Physical Interactions	Vastrik-Stein-2007
**APBB1IP**	BCAR1	0.054839	Physical Interactions	Vastrik-Stein-2007
**SYK**	ITGA2B	0.016105	Physical Interactions	IREF-reactome
**NFYC**	DHCR7	0.051571	Physical Interactions	IREF-reactome
**DHCR24**	DHCR7	0.668485	Physical Interactions	IREF-reactome
**BCAR1**	ITGA2B	0.05653	Physical Interactions	IREF-reactome
**APBB1IP**	ITGA2B	0.046759	Physical Interactions	IREF-reactome
**APBB1IP**	SYK	0.015623	Physical Interactions	IREF-reactome
**APBB1IP**	BCAR1	0.054839	Physical Interactions	IREF-reactome
**# Organism: H. sapiens<br># Application version: 3.6.0<br># Database version: 13 August 2021 00:00:00<br># Network generated on: 22 August 2025<br># Author: GeneMANIA (genemania.org)<br># Notes: Network weight reflects the data source relevance for predicting the function of interest**				

Table S2 *GeneMANIA Output – Networks*

Group	Name	Weight	Reference	PubMed
**Physical Interactions**	Rosenbluh-Hahn-2016	0.281826395	Genetic and Proteomic Interrogation of Lower Confidence Candidate Genes Reveals Signaling Networks in β-Catenin-Active Cancers. Rosenbluh et al. (2016). Cell Syst	http://www.ncbi.nlm.nih.gov/pubmed/27684187
**Physical Interactions**	Bandyopadhyay-Ideker-2010	0.194401743	A human MAP kinase interactome. Bandyopadhyay et al. (2010). Nat Methods	http://www.ncbi.nlm.nih.gov/pubmed/20936779
**Physical Interactions**	IREF-spike	0.148326801		
**Physical Interactions**	Vastrik-Stein-2007	0.033069782	Reactome: a knowledge base of biologic pathways and processes. Vastrik et al. (2007). Genome Biol	http://www.ncbi.nlm.nih.gov/pubmed/17367534
**Physical Interactions**	IREF-reactome	0.033069782		
**Pathway**	REACTOME	0.196362672		
**Genetic Interactions**	Horlbeck-Gilbert-2018 A	0.061952151	Mapping the Genetic Landscape of Human Cells. Horlbeck et al. (2018). Cell	http://www.ncbi.nlm.nih.gov/pubmed/30033366
**Genetic Interactions**	Lin-Smith-2010	0.005266197	A genome-wide map of human genetic interactions inferred from radiation hybrid genotypes. Lin et al. (2010). Genome Res	http://www.ncbi.nlm.nih.gov/pubmed/20508145
**Co-expression**	Wu-Garvey-2007	0.030663552	The effect of insulin on expression of genes and biochemical pathways in human skeletal muscle. Wu et al. (2007). Endocrine	http://www.ncbi.nlm.nih.gov/pubmed/17709892
**Co-expression**	Arijs-Rutgeerts-2009	0.015060925	Mucosal gene expression of antimicrobial peptides in inflammatory bowel disease before and after first infliximab treatment. Arijs et al. (2009). PLoS One	http://www.ncbi.nlm.nih.gov/pubmed/19956723
